# Association between gestational diabetes mellitus and periodontal inflammation in a South African cohort

**DOI:** 10.1007/s00784-026-07213-y

**Published:** 2026-10-10

**Authors:** Mai Ahmed, Dada Oluwaseyi Temilola, Mushi Matjila, Manogari Chetty

**Affiliations:** 1 Department of Craniofacial Biology, Pathology, & Radiology, Faculty of Dentistry, University of the Western Cape, Cape Town, 7500 South Africa; 2 Department of Obstetrics and Gynaecology, University of Cape Town, Cape Town, South Africa

**Keywords:** Gestational diabetes mellitus, Periodontitis, Pregnancy, Inflammation, Bleeding on probing

## Abstract

**Objectives:**

To investigate the association between gestational diabetes mellitus (GDM), periodontal status, and systemic inflammatory gene expression among pregnant women in South Africa.

**Materials and methods:**

This case–control study included 105 pregnant women, comprising 52 women with GDM and 53 normoglycaemic controls. Participants were classified into four groups according to GDM and periodontal status. Periodontal assessment included probing depth (PD), clinical attachment level (CAL), and bleeding on probing (BOP). Peripheral blood expression of CRP, IL-6, TNF-α, MMP-8, and MMP-9 was assessed using quantitative real-time PCR and expressed as relative gene expression. Multivariable logistic regression was adjusted for maternal age, BMI, gestational age, parity, smoking status, ethnicity, and toothbrushing frequency.

**Results:**

PD, CAL, and BOP differed significantly across the four groups (all *p* < 0.001), with the highest BOP observed in women with both GDM and periodontitis. By GDM status, BOP was significantly higher in women with GDM [median 32.5% (range 8–70) vs. 13.0% (range 1–38); *p* < 0.001], whereas PD and CAL did not differ significantly in unadjusted analyses. After adjustment, BOP, PD, and CAL were significantly associated with GDM. MMP-9 relative expression differed overall across the four groups (*p* = 0.047), although adjusted post-hoc pairwise comparisons were not significant. IL-6 differed by GDM status in unadjusted analysis (*p* = 0.039) but not after adjustment.

**Conclusion:**

GDM was associated with greater periodontal inflammatory burden, particularly BOP. Systemic inflammatory gene-expression differences were less consistent and showed no independent associations with GDM after adjustment.

**Supplementary Information:**

The online version contains supplementary material available at https://doi.org/10.1007/s00784-026-07213-y.

## Introduction

Gestational diabetes mellitus (GDM) is the most common metabolic complication of pregnancy and is defined as glucose intolerance first recognised during pregnancy. Its global prevalence has increased substantially in recent decades, driven by rising maternal age, obesity, and lifestyle changes [1, 2]. Current estimates suggest that GDM affects more than 10% of pregnancies worldwide, with particularly high and underreported burdens in low- and middle-income countries, including regions of Africa [2–4]. GDM is associated with significant short- and long-term adverse outcomes, including preeclampsia, macrosomia, neonatal hypoglycaemia, and an increased lifetime risk of type 2 diabetes and cardiovascular disease in both mother and offspring [1].

Systemic inflammation plays a central role in the pathophysiology of GDM. Pregnancy is characterised by physiological insulin resistance, which is further amplified by inflammatory mediators and placental endocrine activity. Elevated levels of pro-inflammatory cytokines, including tumour necrosis factor-α (TNF-α) and interleukins, have been shown to impair insulin signalling and contribute to metabolic dysregulation during gestation [5]. In this context, additional sources of systemic inflammation may further exacerbate metabolic imbalance.

Periodontal diseases, including gingivitis and periodontitis, are chronic inflammatory conditions affecting the tooth-supporting tissues and represent a significant global public health burden. Their prevalence is particularly high in sub-Saharan Africa, where healthcare disparities and limited access to dental services contribute to disease severity [6]. Beyond their local oral effects, periodontal diseases are increasingly recognised as contributors to systemic inflammation and have been associated with diabetes mellitus, cardiovascular disease, and adverse pregnancy outcomes [7].

An expanding body of epidemiological and clinical evidence supports a link between periodontal disease and GDM. Studies have consistently reported a higher incidence of GDM among women with periodontitis, with risk increasing in a severity-dependent manner [8, 9]. Periodontitis may contribute to systemic cytokine elevation, thereby exacerbating insulin resistance during pregnancy. Mechanistic studies further implicate periodontal pathogens, such as *Porphyromonas gingivalis*, and inflammatory mediators, including TNF-α and IL-8, in gestational metabolic dysregulation [8, 9]. Some studies have reported associations between periodontal treatment during pregnancy and GDM-related outcomes; however, evidence regarding a causal or preventive effect remains inconclusive [8].

Despite these advances, important gaps remain. Most available studies have been conducted in high-income or Asian populations, limiting the generalisability of findings to African settings, where both periodontal disease and GDM are prevalent yet under-researched [2, 3, 6]. Evidence simultaneously evaluating clinical periodontal parameters and systemic inflammatory profiles among pregnant women with GDM remains limited [10].

Therefore, the present study aimed to investigate the association between GDM, periodontitis, and systemic inflammatory mediator expression in pregnant women in South Africa. Using a four-group classification (HC, P, GDM, and GDMP), this study further aimed to determine whether systemic inflammatory gene expression differs according to the presence and coexistence of metabolic and periodontal inflammation.

## Materials and methods

### Study design and setting

A case–control analytic study was conducted among 105 pregnant women attending antenatal clinics at Tygerberg and Groote Schuur Hospitals, as well as antenatal clinics in Elsies River, Brackenfell, Chapel Street, and Albow Gardens in Cape Town, South Africa. Participant recruitment and data collection were conducted from April to December 2025 during routine antenatal visits.

Ethical approval was obtained from the Biomedical Research Ethics Committee of the University of the Western Cape (BMREC; reference number BM24/3/11; NHREC registration number REC-130416-050), and the relevant institutional and facility permissions were obtained before participant recruitment. Written informed consent was obtained from all participants before enrolment. The study was conducted in accordance with the Declaration of Helsinki.

### Sample size calculation

The prevalence of periodontitis among women with and without GDM was considered the primary outcome for sample-size estimation. The sample size was calculated using OpenEpi for a case–control study, based on previously reported periodontitis prevalences of 30% among women without GDM and 59% among women with GDM. Using the Fleiss method with continuity correction, a two-sided significance level of α = 0.05, 80% statistical power, and a 1:1 control-to-case ratio, a minimum sample size of 104 participants (52 women with GDM and 52 normoglycaemic controls) was required. The final study sample comprised 105 participants (52 women with GDM and 53 normoglycaemic controls). The sample-size calculation was powered for the primary comparison between women with GDM and normoglycaemic controls. Subsequent stratification into four clinical groups (HC, P, GDM, and GDMP) was undertaken as a secondary exploratory analysis.

### Study population and GDM classification

Pregnant women aged 18–45 years with singleton pregnancies, attending routine antenatal care between 24 and 28 weeks of gestation, and able to provide written informed consent were eligible for inclusion. Pregnant women with GDM and normoglycaemic controls were not individually matched for maternal or behavioural characteristics. Normoglycaemic controls were recruited from the same antenatal population and gestational period as women with GDM. Potential confounding was addressed in the multivariable analyses.

GDM status was determined using a 75-g oral glucose tolerance test (OGTT) performed between 24 and 28 weeks of gestation according to the diagnostic criteria used by the participating antenatal services. Both women with GDM and normoglycaemic controls underwent the same 75-g OGTT, and group allocation was based on the OGTT results.

Exclusion criteria included pre-existing diabetes mellitus; systemic conditions that could influence inflammatory status, including hypertension, human immunodeficiency virus infection, and tuberculosis; antibiotic or anti-inflammatory medication use within the preceding three months; placental, cervical, or uterine abnormalities; periodontal treatment or professional dental cleaning within the preceding six months; active acute dental or periodontal infection or other acute oral inflammatory conditions at the time of examination; and fewer than 18 natural teeth.

### Clinical and demographic data collection

Sociodemographic and clinical information, including maternal age, ethnicity, body mass index (BMI), parity, smoking status, oral hygiene practices, family history of diabetes, and gestational age, was obtained using structured interviewer-administered questionnaires and medical records. BMI was calculated as weight in kilograms divided by height in metres squared (kg/m²).

The periodontal examination and peripheral blood collection were performed during the same antenatal visit between 24 and 28 weeks of gestation, with no intervening study visit between the two procedures, thereby minimizing temporal variation between periodontal assessment and systemic gene-expression measurement.

### Clinical periodontal assessment

All participants underwent a full-mouth periodontal examination performed by the same qualified periodontist using a Williams periodontal probe and a standardized examination protocol. Probing depth (PD), clinical attachment level (CAL), and bleeding on probing (BOP) were recorded at six sites per tooth for all present teeth, excluding third molars.

BOP was recorded 30 s after gentle probing of the gingival sulcus. PD was measured from the gingival margin to the base of the periodontal pocket, and the position of the gingival margin was recorded relative to the cemento-enamel junction (CEJ). CAL was derived from PD and the position of the gingival margin relative to the CEJ and represents the distance from the CEJ to the base of the periodontal pocket. When the gingival margin was located at the CEJ (GM = 0 mm), CAL was numerically equivalent to PD. Periodontal measurements were recorded electronically using Online Periodontal Chart software.

Periodontitis was defined according to the 2017 World Workshop Classification of Periodontal and Peri-Implant Diseases and Conditions as interdental CAL detectable at ≥ 2 non-adjacent teeth, or buccal/oral CAL ≥ 3 mm with pocketing > 3 mm detectable at ≥ 2 teeth, provided that the CAL could not be attributed to non-periodontal causes.

Periodontal stage and grade were not assigned because the data required for complete classification were unavailable. Radiographic bone loss was not assessed during pregnancy, longitudinal disease-progression data were unavailable, and the reasons for tooth loss could not be reliably attributed to periodontitis. Accordingly, participants were classified according to the presence or absence of periodontitis rather than by stage or grade.

Formal intra-examiner reproducibility testing was not performed; therefore, quantitative intra-examiner reliability statistics, including intraclass correlation coefficients (ICC) for PD and CAL, were not available.

### Blood sample collection and RNA processing

Peripheral venous blood samples were collected between 24 and 28 weeks of gestation during the same antenatal visit as the periodontal examination. Whole blood was collected into PAXgene™ Blood RNA tubes for RNA stabilization and subsequently processed for gene-expression analysis.

Total RNA was extracted using the PAXgene Blood RNA Kit in combination with the QIAcube automated extraction system (Qiagen, Germany), according to the manufacturer’s protocol. RNA quantity and quality were assessed using spectrophotometry (NanoDrop 8000), fluorometric quantification, and the Agilent 4200 TapeStation. A quantitative polymerase chain reaction (qPCR)-based SPUD assay using a synthetic DNA control was performed to assess potential PCR inhibition.

Complementary DNA (cDNA) was synthesized from total RNA using LunaScript RT SuperMix. Appropriate negative controls were included to assess contamination and assay performance.

### Quantitative real-time PCR

qPCR was performed to assess peripheral blood gene expression of C-reactive protein (CRP), interleukin-6 (IL-6), tumour necrosis factor-alpha (TNF-α), matrix metalloproteinase-8 (MMP-8), and matrix metalloproteinase-9 (MMP-9).

Gene-specific primers were designed using NCBI Primer-BLAST and subjected to in silico specificity assessment, followed by experimental optimization and validation. Primer annealing temperature and concentration were optimized, and assay specificity, linearity, and amplification efficiency were evaluated before gene-expression analysis. Amplification efficiencies ranged from 98.17% to 101.59%, with R² values ≥ 0.999, consistent with MIQE recommendations.

cDNA was amplified using SYBR Green chemistry on a QuantStudio™ 12 K Flex Real-Time PCR System (Applied Biosystems, USA). Reactions were performed in technical triplicates, with no-template controls included as negative controls. Melt-curve analysis was performed following amplification. Amplification curves were visually inspected, and reactions showing failed amplification, technical-replicate outliers, or evidence of non-specific amplification were excluded from subsequent analysis.

### Primer sequences

Primer sequences and RefSeq accession numbers for the target and candidate reference genes are presented in Table 1.

**Table 1:** Primer sequences used for quantitative real-time PCR

Gene	RefSeq accession	Forward primer (5′→3′)	Reverse primer (5′→3′)
MMP8	NM_002424.3	TCTGACCCTGGTGCCTTGAT	CACAGGGTTTGGGTGTGCTTG
MMP9	NM_004994.3	TTCGACGTGAAGGCGCAGATG	AACTCACGCGCCAGTAGAAGC
CRP	NM_000567.3	TATCACTGGCAGCAGGACGTG	CAGTTCCGTGTAGAAGTGGAGGC
TNF	NM_000594.4	CTGCACTTTGGAGTGATCGGC	GCTACAACATGGGCTACAGGC
IL6	NM_000600.5	AGGGCTCTTCGGCAAATGTAGC	GCCCAGTGGACAGGTTTCTGAC
18 S rRNA	NM_022551.3	GGATGTAAAGGATGGAAAATACA	TCCAGGTCTTCACGGAGCTTGTT
ACTB	NM_001101.5	TGACGTGGACATCCGCAAAG	CTGGAAGGTGGACAGCGAGG
HPRT1	NM_000194.3	GACCAGTCAACAGGGGACAT	CCTGACCAAGGAAAGCAAAG
GAPDH	NM_002046.7	CTACAGCAACAGGGTGGTGGA	GGGTCTCTCTCTTCCTCTTGTGC
IVL	NM_005547.4	GGGTGGTTATTTATGTTTGGGTGG	GCCAGGTCCAAGACATTCAAC
B2M	NM_004048.4	CTCCGTGGCCTTAGCTGTG	TTTGGAGTACGCTGGATAGCCT

### Reference-gene selection and relative gene expression

Six candidate reference genes (18 S rRNA, ACTB, B2M, GAPDH, HPRT1, and IVL) were evaluated for expression stability using RefFinder, which integrates the comparative ΔCt method, geNorm, NormFinder, and BestKeeper algorithms. Based on the stability assessment, relative expression of CRP, IL-6, TNF-α, MMP-8, and MMP-9 was normalized using a combination of four reference genes: GAPDH, HPRT1, 18 S rRNA, and IVL. Relative gene expression was calculated using the Pfaffl method, which accounts for differences in amplification efficiency between target and reference genes, with control samples used as the calibrator.

### Group classification

Participants were classified according to GDM status (GDM vs. non-GDM) and periodontal status (periodontitis vs. no periodontitis). Four study groups were defined: non-GDM without periodontitis (HC), non-GDM with periodontitis (P), GDM without periodontitis (GDM), and GDM with periodontitis (GDMP).

This classification enabled comparison of periodontal clinical parameters and systemic inflammatory gene-expression profiles according to GDM and periodontal status.

### Statistical analysis

Categorical variables were summarized as counts and percentages, while continuous variables were summarized as mean and standard deviation (SD) or median and range, depending on their distribution. Normality of continuous variables was assessed using the Shapiro–Wilk test. Parametric or non-parametric statistical tests were selected according to the distribution of the data. Associations between categorical variables were assessed using the chi-square test or Fisher’s exact test, as appropriate.

For comparisons between two groups, normally distributed continuous variables were analysed using independent-samples *t*-tests, whereas non-normally distributed variables were analysed using the Wilcoxon rank-sum test. Comparisons across the four study groups were performed using one-way analysis of variance (ANOVA) for normally distributed variables and the Kruskal–Wallis test for non-normally distributed variables. Where an overall four-group comparison was statistically significant, post-hoc pairwise comparisons were performed using Dunn’s test for non-parametric analyses, with Bonferroni adjustment for multiple comparisons. In addition to the unadjusted analyses, multivariable logistic regression was performed to evaluate associations with GDM while accounting for potential confounding. GDM status was used as the dependent variable, and models were adjusted for maternal age, BMI, gestational age, parity, smoking status, ethnicity, and toothbrushing frequency as a measure of oral hygiene practice. Each periodontal parameter (PD, CAL, BOP, and periodontitis status) and each inflammatory gene-expression variable were evaluated in separate multivariable logistic regression models to avoid simultaneous inclusion of highly correlated periodontal measures.

Mean PD and mean CAL were modelled per 1-mm increase, while BOP was modelled per 10-percentage-point increase. Relative gene-expression values were log₂-transformed before regression analysis because of their skewed distributions. Accordingly, adjusted odds ratios (aORs) for gene-expression variables represent the change in the odds of GDM associated with a doubling in relative gene expression. aORs with 95% confidence intervals (CIs) were reported.

All statistical tests were two-sided, and statistical significance was set at α = 0.05. Statistical analyses were performed using jamovi version 2.6.44.

## Results

### Participant characteristics

A total of 105 pregnant women were included in the study, comprising 52 women with GDM and 53 normoglycaemic controls. Based on GDM and periodontal status, participants were categorised into four groups: non-GDM without periodontitis (HC, *n* = 25), non-GDM with periodontitis (P, *n* = 28), GDM without periodontitis (GDM, *n* = 18), and GDM with periodontitis (GDMP, *n* = 34).

The median age of the overall cohort was 30 years (range 18–42), the median gestational age was 26 weeks (range 24–28), and the median BMI was 30 kg/m² (range 18–56). Most participants were Black African (67.6%), 61.0% were unemployed, and 74.3% had completed high-school education. A family history of diabetes was reported by 26.7% of participants, and 19.0% were current smokers.

### Comparison across the four study groups

Maternal age (*p* = 0.061), gestational age (*p* = 0.473), and parity (*p* = 0.193) did not differ significantly across the four study groups. In contrast, BMI differed significantly across groups (*p* < 0.001). Ethnic distribution (*p* = 0.021) and smoking status (*p* = 0.047) also differed significantly, whereas family history of diabetes did not differ significantly (*p* = 0.110) (Table 2).

**Table 2:** Baseline characteristics according to gestational diabetes mellitus and periodontal status

A. Demographic and clinical characteristics						
Characteristic	Overall (*n* = 105)	HC (*n* = 25)	P (*n* = 28)	GDM (*n* = 18)	GDMP (*n* = 34)	*p*-value
Age (years)	30 (18–42)	28 (20–40)	29 (18–39)	30.5 (25–38)	31.5 (18–42)	0.061
Gestational age (weeks)	26 (24–28)	26 (24–28)	26 (24–28)	26 (24–28)	27 (24–28)	0.473
BMI (kg/m²)	30 (18–56)	27 (18.5–50)	23 (18–41)	35 (23–54)	36 (18–56)	< 0.001
Parity	2 (0–5)	1 (0–5)	1 (0–4)	2 (0–4)	2 (0–4)	0.193
B. Sociodemographic characteristics						
Characteristic	Overall (*n* = 105)	HC (*n* = 25)	P (*n* = 28)	GDM (*n* = 18)	GDMP (*n* = 34)	*p*-value
Ethnicity, n (%)						0.021
Black African	71 (67.6)	22 (88.0)	19 (67.9)	13 (72.2)	17 (50.0)	
Mixed ancestry	34 (32.4)	3 (12.0)	9 (32.1)	5 (27.8)	17 (50.0)	
Smoking status, n (%)						0.047
Current smoker	20 (19.0)	1 (4.0)	9 (32.1)	2 (11.1)	8 (23.5)	
Non-smoker	85 (81.0)	24 (96.0)	19 (67.9)	16 (88.9)	26 (76.5)	
Family history of diabetes, n (%)						0.110
Yes	28 (26.7)	4 (16.0)	5 (17.9)	8 (44.4)	11 (32.4)	
No	77 (73.3)	21 (84.0)	23 (82.1)	10 (55.6)	23 (67.6)	
C. Periodontal parameters						
Characteristic	Overall	HC	P	GDM	GDMP	*p*-value
PD (mm)	2.6 (1.3–3.7)	1.6 (1.3–3.1)	2.9 (2.1–3.7)	1.95 (1.4–2.6)	2.8 (2.4–3.4)	< 0.001
CAL (mm)	2.6 (1.3–3.7)	1.6 (1.3–3.1)	2.9 (2.1–3.7)	1.95 (1.4–2.6)	2.8 (2.4–3.4)	< 0.001
BOP (%)	20 (1–70)	6 (1–25)	19.5 (8–38)	17.5 (8–27)	41.5 (21–70)	< 0.001
D. Systemic inflammatory gene expression						
Characteristic	Overall	HC	P	GDM	GDMP	*p*-value
CRP relative expression	1.04 (0.09–6.09)	1.05 (0.13–6.09)	1.02 (0.52–1.57)	0.89 (0.19–1.95)	0.93 (0.09–2.60)	0.386
IL-6 relative expression	1.00 (0.28–35.67)	0.99 (0.53–27.86)	0.93 (0.63–1.67)	0.93 (0.47–35.67)	1.09 (0.28–2.53)	0.132
MMP-8 relative expression	1.01 (0.05–10.40)	1.44 (0.12–4.64)	0.98 (0.05–10.40)	0.71 (0.19–8.77)	1.45 (0.09–8.22)	0.731
MMP-9 relative expression	0.91 (0.06–5.71)	0.71 (0.14–3.74)	1.61 (0.06–5.71)	0.62 (0.30–4.07)	0.77 (0.12–4.94)	0.047
TNF-α relative expression	1.06 (0.20–1.90)	1.04 (0.33–1.60)	1.06 (0.55–1.86)	1.04 (0.20–1.75)	1.12 (0.71–1.90)	0.250

Continuous variables are presented as median (range), and categorical variables as number (percentage). HC, non-GDM without periodontitis; P, non-GDM with periodontitis; GDM, GDM without periodontitis; GDMP, GDM with periodontitis. Statistical significance was set at *p* < 0.05

**Fig. 1:**
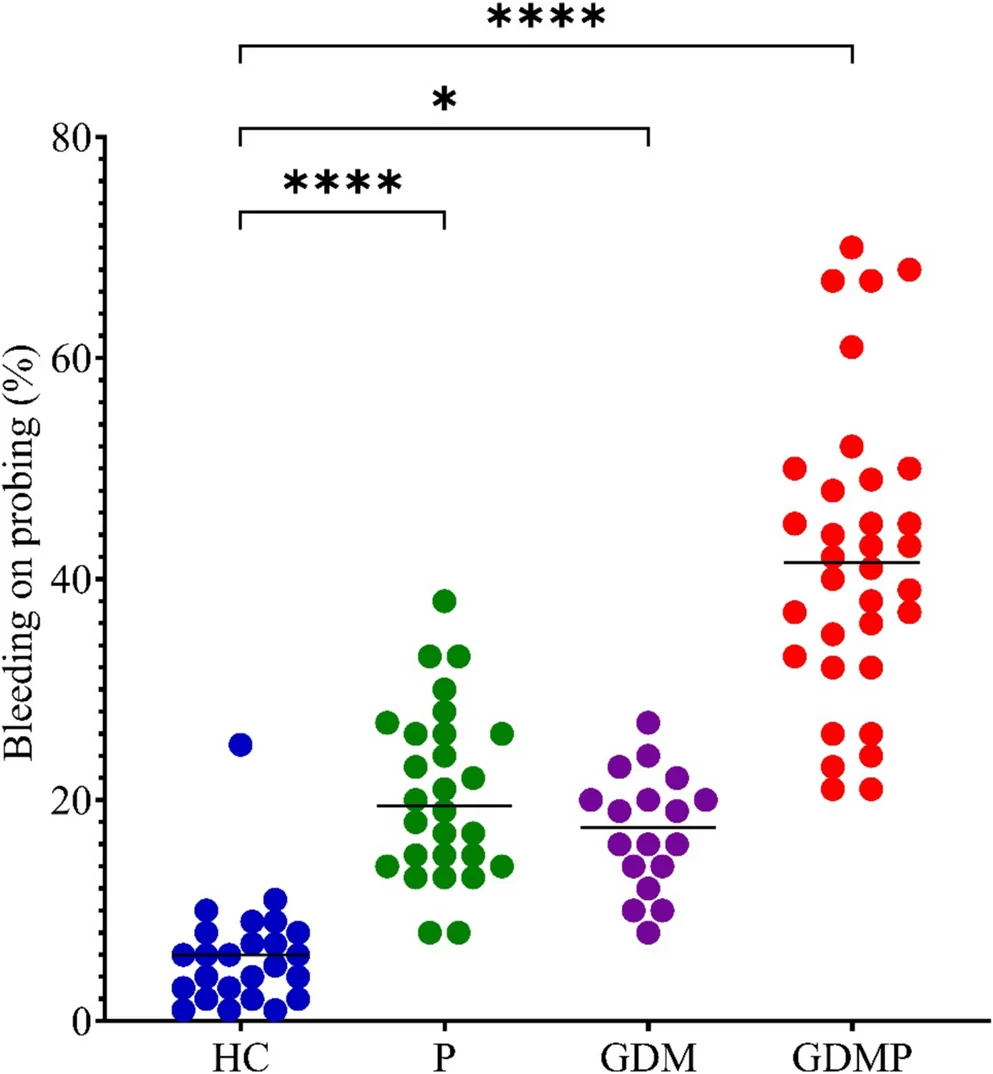
Bleeding on probing across study groups. Individual participant values are shown, with horizontal lines indicating group medians. Differences between groups were assessed using the Kruskal–Wallis test followed by Dunn’s multiple-comparisons test with Bonferroni adjustment. *p* < 0.05; *p < 0.0001

All three periodontal clinical parameters differed significantly across the four groups. Median PD was 1.6 mm in HC, 2.9 mm in P, 1.95 mm in GDM, and 2.8 mm in GDMP (*p* < 0.001). Median CAL was 1.6 mm, 2.9 mm, 1.95 mm, and 2.8 mm, respectively (*p* < 0.001). Bleeding on probing (BOP) also differed significantly across groups, with median values of 6.0% in HC, 19.5% in P, 17.5% in GDM, and 41.5% in GDMP (*p* < 0.001). The highest BOP was observed in the GDMP group (Fig. 1).

CRP, IL-6, TNF-α, and MMP-8 relative gene expression did not differ significantly across the four study groups. In contrast, MMP-9 relative gene expression differed significantly across the four groups (*p* = 0.047). Median MMP-9 relative expression was 0.71 in HC, 1.61 in P, 0.62 in GDM, and 0.77 in GDMP. However, Bonferroni-adjusted post-hoc pairwise comparisons did not identify statistically significant differences between individual group pairs.

### Comparison according to gestational diabetes status

Women with GDM were significantly older than normoglycaemic women (median 31 vs. 28 years; *p* = 0.010), had a higher BMI (median 35.5 vs. 24.0 kg/m²; *p* < 0.001), and had higher parity (*p* = 0.030). A family history of diabetes was also more frequent among women with GDM than among normoglycaemic participants (36.5% vs. 17.0%; *p* = 0.041). Gestational age did not differ significantly between the groups (*p* = 0.620) (Table 3).

**Table 3:** Characteristics according to gestational diabetes mellitus status

Characteristic	GDM (*n* = 52)	Non-GDM (*n* = 53)	*p*-value
Age (years)	31 (18–42)	28 (18–40)	0.010
Gestational age (weeks)	27 (24–28)	26 (24–28)	0.620
BMI (kg/m²)	35.5 (18–56)	24.0 (18–50)	< 0.001
Parity	2 (0–4)	1 (0–5)	0.030
Family history of diabetes, n (%)	19 (36.5)	9 (17.0)	0.041
PD (mm)	2.65 (1.4–3.4)	2.30 (1.3–3.7)	0.118
CAL (mm)	2.65 (1.4–3.4)	2.30 (1.3–3.7)	0.116
BOP (%)	32.5 (8–70)	13.0 (1–38)	< 0.001
CRP relative expression	0.92	1.05	0.087
IL-6 relative expression	1.08	0.95	0.039
MMP-8 relative expression	0.99	1.02	0.728
MMP-9 relative expression	0.73	1.07	0.269
TNF-α relative expression	1.10	1.05	0.284

Continuous variables are presented as median (range), where applicable. The identical displayed PD and CAL summary values reflect rounding; the underlying participant-level measurements were not completely identical

In the unadjusted analysis, PD (*p* = 0.118) and CAL (*p* = 0.116) did not differ significantly according to GDM status. In contrast, BOP was significantly higher among women with GDM than among normoglycaemic participants [32.5% (8–70) vs. 13.0% (1–38); *p* < 0.001].

IL-6 relative gene expression differed significantly according to GDM status in the unadjusted analysis (*p* = 0.039). No statistically significant differences were observed for CRP (*p* = 0.087), MMP-8 (*p* = 0.728), MMP-9 (*p* = 0.269), or TNF-α (*p* = 0.284).

### Comparison according to periodontal status

Participants with periodontitis had significantly greater PD, CAL, and BOP than those without periodontitis (all *p* < 0.001) (Table 4). Smoking was also more frequent among participants with periodontitis.

**Table 4:** Characteristics according to periodontal status

Characteristic	No periodontitis (*n* = 43)	Periodontitis (*n* = 62)	*p*-value
PD (mm)	1.7 (1.3–3.1)	2.9 (2.1–3.7)	< 0.001
CAL (mm)	1.7 (1.3–3.1)	2.9 (2.1–3.7)	< 0.001
BOP (%)	9.0 (1–27)	31.0 (8–70)	< 0.001
CRP relative expression	1.04	0.97	0.636
IL-6 relative expression	0.95	1.03	0.850
MMP-8 relative expression	0.97	1.02	0.634
MMP-9 relative expression	0.68	1.07	0.060
TNF-α relative expression	1.04	1.10	0.195

No statistically significant differences in systemic inflammatory gene expression were observed according to periodontal status for CRP (*p* = 0.636), IL-6 (*p* = 0.850), TNF-α (*p* = 0.195), MMP-8 (*p* = 0.634), or MMP-9 (*p* = 0.060).

### Association Between GDM and Periodontitis

**Fig. 2:**
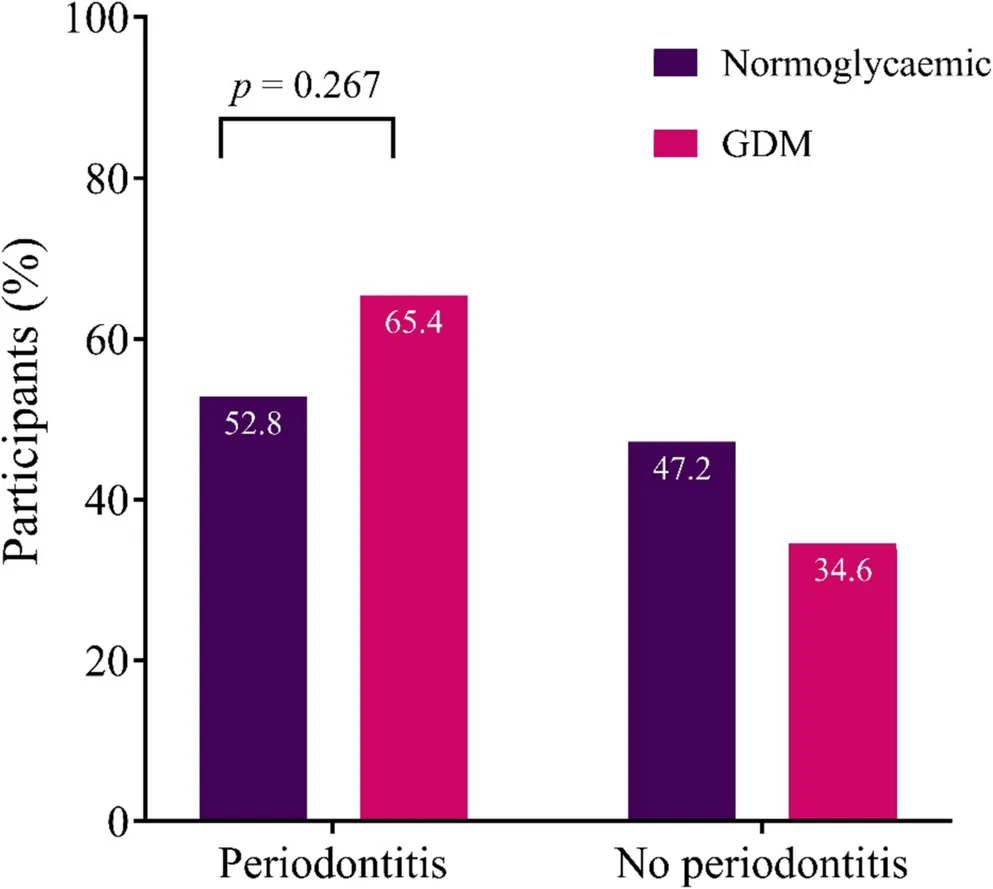
Prevalence of periodontitis according to gestational diabetes mellitus status. Periodontitis was observed in 65.4% of women with GDM and 52.8% of normoglycaemic women. The difference was not statistically significant (*p* = 0.267)

 Periodontitis was present in 34 of 52 women with GDM (65.4%) and 28 of 53 normoglycaemic participants (52.8%). Although periodontitis was more frequent among women with GDM, this difference was not statistically significant (*p* = 0.267) (Fig. 2).

### Multivariable analysis

Multivariable logistic regression analyses were performed to evaluate associations with GDM after adjustment for maternal age, BMI, gestational age, parity, smoking status, ethnicity, and toothbrushing frequency (Table 5).

**Table 5:** Multivariable logistic regression analysis of factors associated with gestational diabetes mellitus

Variable	aOR	95% CI	*p*-value
Mean PD, per 1-mm increase	2.35	1.06–5.22	0.036
Mean CAL, per 1-mm increase	2.35	1.06–5.21	0.035
BOP, per 10-percentage-point increase	4.62	2.31–9.24	< 0.001
Periodontitis status	1.96	0.75–5.15	0.170
CRP, per doubling of relative expression	0.63	0.38–1.05	0.077
IL-6, per doubling of relative expression	1.15	0.71–1.87	0.557
MMP-8, per doubling of relative expression	1.06	0.80–1.41	0.668
MMP-9, per doubling of relative expression	0.99	0.71–1.39	0.964
TNF-α, per doubling of relative expression	1.32	0.54–3.23	0.547

Models were adjusted for maternal age, BMI, gestational age, parity, smoking status, ethnicity, and toothbrushing frequency. Gene-expression variables were log₂-transformed; therefore, the corresponding aORs represent the change in the odds of GDM associated with a doubling in relative gene expression. aOR, adjusted odds ratio; CI, confidence interval

BOP remained strongly associated with GDM after adjustment. Each 10-percentage-point increase in BOP was associated with higher odds of GDM (aOR 4.62, 95% CI 2.31–9.24; *p* < 0.001). Mean PD was also associated with GDM after adjustment, with each 1-mm increase associated with higher odds of GDM (aOR 2.35, 95% CI 1.06–5.22; *p* = 0.036). Similarly, each 1-mm increase in mean CAL was associated with higher odds of GDM (aOR 2.35, 95% CI 1.06–5.21; *p* = 0.035). Periodontitis status was not significantly associated with GDM after adjustment (aOR 1.96, 95% CI 0.75–5.15; *p* = 0.170).

None of the systemic inflammatory gene-expression variables was significantly associated with GDM after adjustment. The significant difference in IL-6 relative gene expression observed in the unadjusted analysis was no longer significant after adjustment (aOR 1.15, 95% CI 0.71–1.87; *p* = 0.557).

## Discussion

This study demonstrates that GDM is associated with increased periodontal inflammation among pregnant women in South Africa. To our knowledge, this is one of the first studies from a South African population to examine the relationship between GDM, periodontal inflammation, and systemic inflammatory gene expression. These findings contribute to the limited evidence on this association in African populations.

Women with GDM exhibited markedly higher BOP compared with normoglycaemic controls, indicating a greater periodontal inflammatory burden associated with GDM during pregnancy. Although PD did not differ significantly between women with and without GDM in the unadjusted analysis, it was significantly associated with GDM after adjustment for maternal age, BMI, gestational age, parity, smoking status, ethnicity, and toothbrushing frequency. When periodontal and metabolic status were considered jointly, participants with both GDM and periodontitis exhibited the greatest periodontal inflammatory burden, particularly reflected by BOP. BOP is a clinical indicator of current gingival inflammatory activity and may therefore be particularly sensitive to the inflammatory changes occurring during pregnancy [11].

Importantly, CAL did not differ significantly between women with and without GDM in the unadjusted analysis but was significantly associated with GDM after multivariable adjustment. This finding should be interpreted cautiously. CAL reflects cumulative periodontal tissue destruction and may represent a history of periodontal disease, whereas BOP more directly reflects current gingival inflammatory status [11, 12]. Pregnancy-related hormonal and immunological changes may intensify gingival inflammation without necessarily producing corresponding increases in clinical attachment loss during pregnancy [13]. Furthermore, because of the case–control design, the temporal relationship between attachment loss and GDM cannot be established; therefore, the adjusted CAL association should not be interpreted as evidence that GDM contributed to periodontal attachment loss. In contrast, BOP showed the strongest independent association with GDM among the periodontal parameters evaluated, supporting an association between GDM and greater periodontal inflammatory burden.

The association between GDM and periodontitis is biologically plausible. Hyperglycaemia promotes immune dysregulation, oxidative stress, and endothelial dysfunction, leading to exaggerated inflammatory responses in periodontal tissues [14]. The formation of advanced glycation end-products (AGEs) and their interaction with the receptor for AGEs (RAGE) further amplify pro-inflammatory responses and cytokine production [15].

**Fig. 3:**
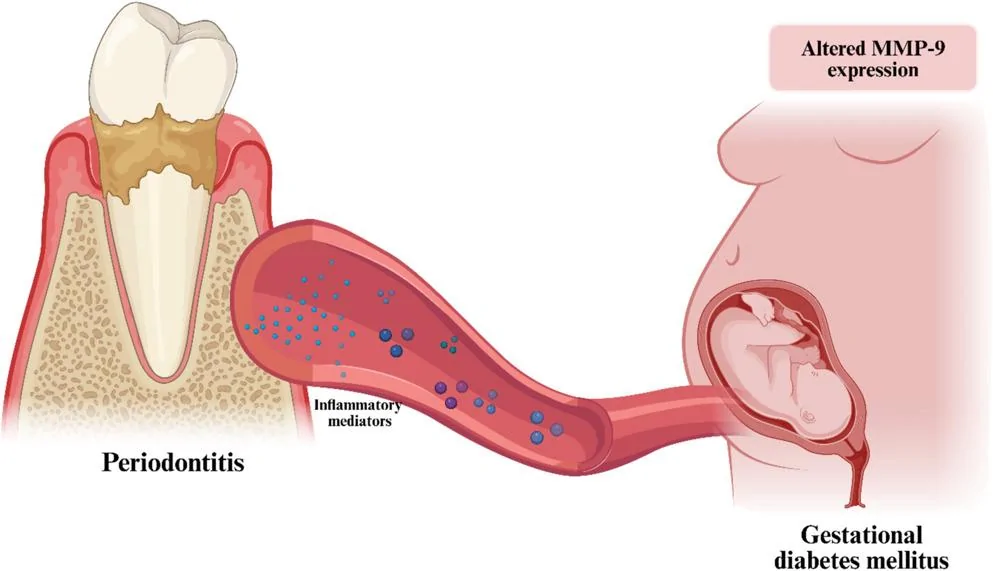
Conceptual illustration of the association between periodontitis and gestational diabetes mellitus. Periodontal and metabolic inflammation may contribute to overlapping systemic inflammatory pathways during pregnancy. Differences in inflammatory gene expression may reflect context-dependent systemic responses associated with these conditions. The proposed pathways represent biologically plausible associations and do not imply causality

 During pregnancy, these mechanisms are compounded by physiological insulin resistance and placental secretion of pro-inflammatory mediators, resulting in a heightened systemic inflammatory environment [16]. This may explain the significantly higher BOP observed among women with GDM, with periodontal tissues potentially reflecting increased inflammatory susceptibility. However, because of the case–control design, the direction of this relationship cannot be established, and the findings should be interpreted as an association rather than evidence that GDM causes periodontal inflammation. A conceptual overview of this relationship, highlighting potential overlapping inflammatory pathways between GDM and periodontitis, is presented in Fig. 3.

Our findings are consistent with previous epidemiological and clinical investigations reporting an association between periodontal disease and GDM. Several case–control and cohort studies have demonstrated increased periodontitis in women with GDM, particularly in relation to PD and BOP [9, 17, 18]. Meta-analyses further support a relationship between periodontal disease and increased GDM risk, with severity-dependent effects [19]. However, most existing evidence originates from high-income or Asian populations, limiting generalisability to African settings [20]. This study contributes important data from a South African population, where both periodontal disease and metabolic disorders remain under-researched. The findings therefore extend current evidence to a population with distinct socioeconomic and healthcare contexts [20]. The absence of a significant unadjusted difference in CAL between women with and without GDM may reflect the distinction between pregnancy-related active gingival inflammation and cumulative periodontal tissue destruction [13, 21].

Despite clear differences in clinical periodontal inflammation, systemic inflammatory gene expression showed limited differentiation according to GDM or periodontal status. This may reflect pregnancy-associated immune adaptation, whereby maternal immune responses are tightly regulated to maintain fetal tolerance [22, 23]. Periodontal inflammation originates locally, while its systemic effects depend on the dissemination of inflammatory mediators, microbial components, and activated immune cells into the circulation; therefore, local inflammatory activity may not be directly reflected by peripheral blood gene-expression measurements [24]. In the unadjusted analysis, IL-6 relative gene expression differed significantly according to GDM status; however, this association was attenuated and was no longer statistically significant after adjustment for maternal age, BMI, gestational age, parity, smoking status, ethnicity, and toothbrushing frequency. CRP, TNF-α, MMP-8, and MMP-9 were also not independently associated with GDM after adjustment. Previous studies have reported alterations in CRP, IL-6, TNF-α, MMP-8, and MMP-9 in GDM and/or periodontal disease, although findings vary according to the population studied, biological sample, and analytical approach [17, 18, 25].

Across the four study groups, MMP-9 relative gene expression showed a significant overall difference. However, Bonferroni-adjusted post-hoc comparisons did not identify significant differences between individual group pairs. Therefore, the overall MMP-9 finding should be interpreted cautiously and considered exploratory, particularly given the smaller subgroup sizes and the reduced statistical power associated with multiple-comparison adjustment. The finding should not be attributed to any specific study group, and larger studies are needed to determine whether the observed variation in MMP-9 expression across GDM and periodontal groups is reproducible.

The lack of significant adjusted associations for IL-6 and TNF-α may reflect differences between local periodontal and systemic inflammatory responses. Pregnancy is characterized by substantial immunological adaptation, and inflammatory mediator profiles in the systemic circulation may not necessarily parallel inflammatory activity within periodontal tissues [10, 22, 23]. Furthermore, peripheral blood gene expression provides a systemic measure that may not fully capture tissue-specific or temporally dynamic inflammatory responses during pregnancy. Future longitudinal studies incorporating both peripheral blood and local periodontal samples, such as gingival crevicular fluid, may provide a more comprehensive understanding of the relationship between periodontal and systemic inflammation in GDM.

The present study assessed inflammatory mediators at the gene-expression level because its primary molecular objective was to evaluate systemic transcriptional inflammatory responses associated with GDM and periodontal status. Peripheral blood RNA was collected and stabilised specifically for gene-expression analysis, enabling the assessment of transcriptional changes in the selected inflammatory markers. However, gene-expression measurements reflect transcriptional activity rather than circulating protein concentrations, and mRNA abundance does not necessarily correspond directly to functional protein abundance because of post-transcriptional and translational regulatory mechanisms [26]. Therefore, the present findings reflect transcriptional activity and should not be interpreted as direct evidence of differences in circulating CRP, IL-6, TNF-α, MMP-8, or MMP-9 protein concentrations. Future studies combining gene-expression and protein-level measurements may provide a more comprehensive assessment of systemic inflammatory activity.

BMI was significantly higher among women with GDM, consistent with its established role in metabolic dysregulation [5]. Obesity represents a chronic pro-inflammatory state that may contribute to both systemic and periodontal inflammation [5, 24]. Importantly, the present study incorporated multivariable analyses adjusting for BMI together with maternal age, gestational age, parity, smoking status, ethnicity, and toothbrushing frequency. Following adjustment, BOP, PD, and CAL remained significantly associated with GDM, whereas periodontitis status itself was not independently associated with GDM. Nevertheless, residual confounding from unmeasured factors cannot be excluded.

Ethnicity and smoking differed significantly across the four periodontal–GDM groups. The higher proportion of mixed ancestry participants in the GDM with periodontitis group may reflect differences in broader socioeconomic, behavioural, environmental, and structural determinants of health rather than intrinsic biological susceptibility [27–29]. Smoking, a well-established risk factor for periodontal disease, was more prevalent among participants with periodontitis and may have contributed to the observed findings [30]. Importantly, oral hygiene practices did not differ significantly across groups, indicating that differences in the measured oral hygiene practices were unlikely to fully explain the observed periodontal findings [24]. However, these observations should be interpreted cautiously because ethnicity may represent multiple interacting social, environmental, behavioural, and healthcare-related influences rather than an independent biological determinant [27–29].

An important contribution of the present study is the simultaneous evaluation of GDM status, clinical periodontal parameters, and peripheral blood inflammatory gene expression in a South African pregnant population. Evidence integrating these clinical and molecular measures in African populations remains limited. By evaluating periodontal and systemic inflammatory characteristics within the same cohort, this study provides additional insight into the relationship between metabolic and periodontal inflammatory burden during pregnancy.

From a public health perspective, the observed association between GDM and greater periodontal inflammatory burden highlights the potential value of oral health assessment during pregnancy. Periodontal evaluation may help identify pregnant women with increased oral inflammatory burden and facilitate appropriate dental referral and management [31, 32]. This may be particularly relevant in low- and middle-income settings where unmet oral healthcare needs remain substantial [31, 33, 34]. However, the present findings do not establish that periodontal assessment can predict GDM or that periodontal treatment can reduce GDM risk or improve metabolic or pregnancy outcomes. Therefore, firm recommendations regarding the integration of periodontal screening into routine antenatal care cannot be made until these associations are confirmed in larger prospective and interventional studies.

We acknowledge several limitations of this study. The case–control design precludes causal inference, and the observed relationships should therefore be interpreted as associations. Although multivariable analyses adjusted for maternal age, BMI, gestational age, parity, smoking status, ethnicity, and toothbrushing frequency, residual confounding from unmeasured factors cannot be excluded. Radiographic assessment was not performed during pregnancy, limiting the evaluation of cumulative periodontal destruction. In addition, longitudinal disease-progression data and reliable information regarding reasons for tooth loss were unavailable, preventing comprehensive periodontal staging and grading. Although all periodontal examinations were performed by the same periodontist using a standardized protocol, formal intra-examiner reproducibility testing was not performed. Consequently, quantitative reliability measures for PD and CAL were unavailable, which should be considered when interpreting the reproducibility of these measurements. Future studies should incorporate formal intra-examiner reliability assessment. Inflammatory gene expression was assessed in peripheral blood and may not fully reflect local inflammatory processes within periodontal tissues. Furthermore, gene expression was assessed at the mRNA level rather than by measuring circulating protein concentrations, and mRNA abundance does not necessarily correspond to functional protein abundance because of post-transcriptional regulation, mRNA stability, translational control, protein degradation, and post-translational modifications [26]. Finally, the original sample-size calculation was based on the primary comparison of periodontitis prevalence between women with and without GDM and was not specifically powered for the secondary four-group gene-expression analyses. Consequently, the smaller subgroup sizes may have limited the ability to detect modest differences in inflammatory gene expression, particularly after correction for multiple comparisons.

## Conclusion

GDM was associated with a greater periodontal inflammatory burden, with BOP demonstrating the strongest and most consistent association. PD and CAL were also associated with GDM after adjustment for relevant maternal and behavioural confounders, whereas the presence of periodontitis itself was not independently associated with GDM. Systemic inflammatory gene-expression findings were less consistent: MMP-9 gene expression showed a significant overall difference across the four study groups, although post-hoc pairwise comparisons were not significant, and IL-6 gene expression differed by GDM status in the unadjusted analysis but was not independently associated with GDM after multivariable adjustment. These findings highlight the distinction between clinical periodontal inflammation and peripheral blood inflammatory gene expression during pregnancy and support further investigation in larger prospective studies.

## Supplementary Information

Below is the link to the electronic supplementary material.

Supplementary Material 1

## Data Availability

The datasets generated and/or analysed during the current study are available from the corresponding author on reasonable request. Due to ethical and privacy considerations, the data are not publicly available.

## Declarations

### Generative AI and AI-Assisted technologies in the manuscript preparation process

During the preparation of this manuscript, the authors used AI-assisted tools to improve language and readability. The authors reviewed and edited the content and take full responsibility for the final manuscript.
